# Effect of Vitamin B6 Versus Propranolol on Antipsychotic-Induced Akathisia: A pilot Comparative Double-blind Study

**Published:** 2018

**Authors:** Narges Shams-Alizadeh, Hamid Bakhshayesh, Farzin Rezaei, Ebrahim Ghaderi, Nasim Shams-Alizadeh, Kambiz Hassanzadeh

**Affiliations:** a *Department of Psychiatry, Faculty of Medicine, Kurdistan University of Medical Sciences, Sanandaj, Iran.*; b *Social Determinants of Health Research Center, Kurdistan University of Medical Sciences, Sanandaj, Iran.*; c *Lifestyle modification research center, Imam Reza hospital, Kermanshah University of Medical Sciences, Kermanshah, Iran. *; d *Cellular and Molecular Research Center, Kurdistan University of Medical Sciences, Sanandaj, Iran.*; e *Department of Physiology and Pharmacology, Faculty of Medicine, Kurdistan University of Medical Sciences, Sanandaj, Iran.*

**Keywords:** Antipsychotic, Akathisia, Clinical trial, Propranolol, Vitamin B6

## Abstract

Akathisia is a common adverse effect of antipsychotic drugs and is characterized by subjective feelings of restlessness. First-line treatment usually consists of propranolol, a beta adrenergic antagonist. However, propranolol does not seem to be efficacious in up to 70% of patients. This study was aimed to evaluate the effect of vitamin B6 versus propranolol on antipsychotic-induced akathisia (AIA). This study was a comparative, double-blind, randomized trial. In the present study, 66 adult patients with antipsychotic-induced akathisia were enrolled and randomized into three groups, and received vitamin B6 300 mg/12 h or 600 mg/12 h or propranolol 20 mg/12 h. The diagnosis of AIA was made by clinical examination and its severity was assessed by the Barnes Akathisia Rating Scale.

Fifty one patients completed 5 days of the trial. The results showed that there was no significant difference in BARS score among the different groups which means that vitamin B6 attenuated the AIA similar to propranolol. However, there wasn’t any significant difference between high or low dose of vitamin B6. In conclusion, the results of this trial suggest that vitamin B6 may be beneficial for ameliorating of antipsychotic-induced akathisia.

## Introduction

Akathisia is one of the most distressing side effects of antipsychotic drugs (1). The diagnosis requires characteristic restless movements and typical subjective complaints of restlessness referable to the legs, inner tension, and discomfort. Akathisia is reported to be the most common reason cited by patients for discontinuing their medications (2). 

Subtypes of akathisia have been described which include acute, chronic, tardive, and withdrawal akathisia (3). The frequency of acute akathisia varies from 8% to 76%, while that of chronic or tardive akathisia varies from 0.1% to 41% (4).

 The neurobiological mechanisms are responsible for the development of akathisia remains unclear, with various theories suggested. It is suggested that an imbalance of several neurotransmitter systems (such as dopamine, norepinephrine, acetylcholine, F-amino butyric acid, and serotonin) in tegmental and nigrostriatal areas may be involved in the pathogenetic process (5).

 In order to control the antipsychotic-induced akathisia (AIA) usually antipsychotic drug regimens are modified in addition to anti-akathisia agent such as β-adrenergic and 5-HT2 blockers are added to this regimens (6). In fact the first-line therapy usually consists of propranolol, a beta-adrenergic antagonist. Propranolol, however, does not seem to be efficacious in up to 70% of patients; it has poor tolerability, and its use is limited in patients with bronchial asthma, diabetes mellitus, hypotension, or conduction block (6). Serotonin 2A (5-HT2A) antagonists have been suggested as anti-akathisia agents, according to the hypothesis that the low affinity of second generation anti-psychotics to induce extrapyramidal side effects is accounted for, at least in part, by the preponderance of the 5-HT2A receptor antagonism over the dopamine D2 receptor blockade (7). In this regard, studies indicated that non-selective post-synaptic 5-HT2 blockers such as ritanserin, cyproheptadine, mianserin, mirtazapine, and trazodone were anti-akathisic effect and are currently utilize as second-line treatments for akathisia (8, 9). Other drugs such as anticholinergics, benzodiazepines, amantadine, clonidine, and dopamine agonists have been used to manage this adverse effect, but they are efficacious in only a minority of patients (6).

 On the other hand, vitamin B6 is reported to be effective in the treatment of movement disorders induced by various psychotropic agents (10-13). Previous studies have described patients with tardive movement disorders, lithium-induced tremor, and only a small number with neuroleptic-induced akathisia. This suggests that vitamin B6 may also be an option in the treatment of acute akathisia therefor this study was aimed to compare the effect of pyridoxine and propranolol on AIA in a randomized clinical trial. 

## Methods

*Participants*

Sixty six individuals diagnosed with antipsychotic-induced akathisia (18-50 years of age) were recruited to the study. A sample size of 20 patients for vitamin B6 300 mg/12 h, 20 patients for 600 mg/12 h and 20 patients for propranolol 20 mg/12 h was obtained by the formula. The block randomization method was designed to randomize the subjects into three groups. It is worth noting that the groups received their routine treatment in addition to the propranolol or vitamin B6. Patients were excluded if treatment with anticholinergic agents had been initiated less than 10 days before screening. Furthermore, patients were receiving adrenergic receptor antagonists and/or every vitamin therapy was excluded. 

**Table 1.** Basic characteristic of the participants

**Criteria**	**Propranolol (n = 17)**	**Vit B6 600 mg (n = 17) Vit B6 1200 mg (n=17)**	**P value**
Age (year, mean ± SD)	(33 ± 15)	(38 ± 14) (35 ±10)	0.82
Sex (Male/Female)	(12, 5) (%70.6, %29.4)	(10, 7) (12, 5) (%58.8, %41.2) (%70.6,%29.4)	0.76
BARS score (mean ± SD)	(5.7 ± 1.76)	(5.76 ± 1.39) (6 ±1.37)	0.8

**Table 2:** The participants diagnosis in different groups

**Diagnosis (n, %)**	**Propranolol (n = 17)**	**Vit B6 600 mg (n = 17) Vit B6 1200 mg (n=17)**
Schizophrenia	(4, 23.5)	(3, 17.6 ) (6, 35.3)
Schizoaffective disorders	(2, 11.8)	(1, 5.9) (2, 11.8)
Psychosis	(2, 11.8)	(4, 23.5) (2, 11.8)
Bipolar disorder	(9, 52.9)	(6, 35.3) (5, 29.4)
Other disorder	(0, 0)	(3, 17.7) (2, 11.8)
Total	(17, 100)	(17, 100) (17, 100)

**Table 3:** The type of medications used by the study participants

**Medication (n, %)**	**Propranolol (n = 17)**	**Vit B6 600 mg (n = 17) Vit B6 1200 mg (n=17)**	**P value**
Typical antipsychotic	(5, 29.4)	(11, 64.7) (8, 47.1) 0.11	
Atypical antipsychotic	(14, 82.4)	(13, 76.5) (16, 94.1)	0.29
Anticholinergic	(13, 76.5)	(13, 76.5) (13, 76.5)	0.97
Mood stabilizer	(12, 70.6)	(10, 58.8) (10, 58.8)	0.7

**Figure 1:**
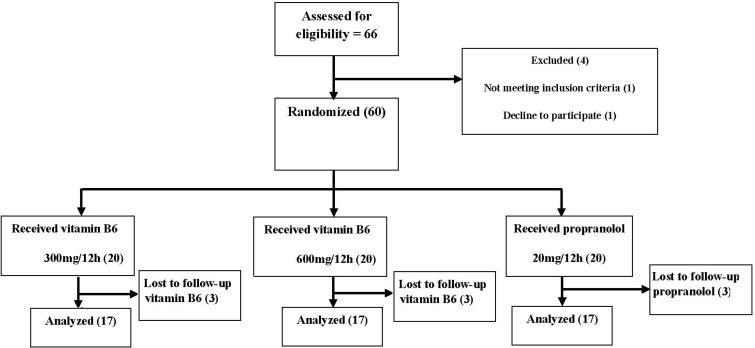
Trial flow diagram

**Figure 2:**
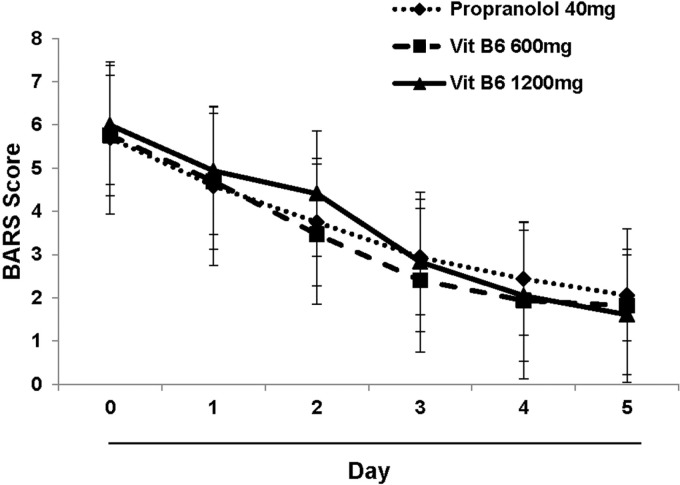
Changes in akathisia level during the study period. The mean BARS scores at day 0 (baseline) and five consecutive days are displayed. Each points indicates the mean ± SD. *P* *value* less than 0.05 is considered significant. BARS: Barnes Akathisia Rating Scale

*Study design*

The present study was done at a single site, the Ghods hospital in Sanandaj. The protocol was approved by the Ethics Committee of Kurdistan University of Medical Sciences and the clinical trial was registered in the Iranian Registry of Clinical Trials (IRCT) with code number of (IRCT201111042935). The procedures used in this trial were conducted in accordance with international ethical standards and written informed consent was obtained from all participants after a full explanation of the nature of the study and its protocol. 

The study was a double-blind, comparative randomized clinical trial of administration of vitamin B6 or propranolol in AIA admitted to the Ghods hospital. Participants received either vitamin B6 300 mg/12 h (N = 17) or 600 mg/12 h (N = 17) or propranolol 20 mg/12 h were purchased from Sobhan Company (Tehran, Iran) for 5 consecutive days. The doses of all medications remained unchanged for at least 3 days before the baseline ratings and during the entire study period of 5 days and the preparations were available in identical capsules. At randomization, oral vitamin B6 was initiated at 600 or 1200 mg/d and all patients received the target doses. The above doses were chosen based on previous studies that were used for treating movement disorders induced by various psychotropic agents (10-13). 

*Tools and outcome evaluation*

Subjects -recruited from inpatients with schizophrenia were screened for AIA admitted to the Ghods hospital; an academic hospital affiliated to the Kurdistan University of Medical Sciences. The diagnosis of AIA was made by clinical examination and its severity was assessed with the Barnes Akathisia Rating Scale (BARS) (14, 15). The BARS is the most widely used rating scale for akathisia. It is a rating scale for drug-induced akathisia that incorporates diagnostic criteria for pseudoakathisia, and for mild, moderate, and severe akathisia. It comprises items for rating the observable, restless movements which characterise the condition, the subjective awareness of restlessness, and any distress associated with the akathisia. In addition, there is an item for rating global severity (14).

This standard tool has been used and known in previous Iranian studies as a valid Persian version of BARS to rate the severity of akathisia (16). The criteria for patient recruitment were: ages between 18 and 50 years; currently on antipsychotic medication; a score of at least 2 (mild akathisia) on the global subscale of the Barnes Akathisia Rating Scale (BARS); no history of restless legs syndrome, Parkinson’s disease, peripheral neuropathy, peripheral vascular disease, diabetes mellitus, head trauma, or drug dependence. 

*Data analysis*

All results are presented as mean ± SD. P-value less than 0.05 was considered significant. Repeated measures analysis of variances (ANOVA) was used to analyze the differences among group means and Chi-square was used to compare the observed clinical symptoms.

## Results

Fifty one patients who met our inclusion criteria were randomly assigned to receive, in a double-blind manner, propranolol (n=17) or vitamin B6 300 mg/12 h (n=17) or vitamin B6 600 mg/12 h (n=17). All the patients completed the study (Figure 1).

Data regarding demographic characteristics and clinical variables of participants is summarized in Table 1. The groups showed similar values in their Male/Female ratio and age. Likewise, there were no significant basal (day 0) differences among the groups in the BARS scores.

Comparing the diagnosis of participants in different groups, we found no significant differences in the diagnosis among different groups of the present study (Table 2). Moreover, our findings revealed that there was no significant difference among the groups regarding the type of medications (e.g. typical or atypical antipsychotic, anticholinergic or mood stabilizer) used by the study participants (Table 3).

Medication adherence was assessed by capsule count. The percentage of capsules taken was measured by the number of consumed capsules each day divided by the total number of capsules prescribed. All groups revealed good adherence. The medication adherence was 100%. In fact, no patient returned any capsule at the final visit. Therefore, there was no significant difference among groups in the mean percentage of prescribed capsules taken by the participants.

As depicted in Figure 2. there is a similar decreasing trend in BARS score in propranolol and vitamin B6 (both doses) received subjects. The data analysis indicated that there is no significant difference in BARS score among the different study groups which means that vitamin B6 ameliorated the akathisia symptoms induced by antipsychotic agents similar to propranolol. However, there wasn’t any significant difference between high or low dose of vitamin B6.

## Discussion

To our knowledge, the results of the present study for the first time showed that the effect of vitamin B6 on AIA was as effective as propranolol; currently available therapeutic regimen for akathisia. However, in the present trial using two doses of vitamin B6, there did not appear to be a significant difference between these two doses. Therefore, vitamin B6 may serve as an alternative option in the treatment of antipsychotic induced akathisia in patients with schizophrenia. 

It is well documented that development of akathisia has a negative impact on long-term treatment outcomes in patients with schizophrenia (17, 18). Although therapeutic agents such as beta-adrenergic blockers, benzodiazepines, and anticholinergic drugs have been routinely used for ameliorating the akathisia, they show only a moderate efficacy, and a large proportion of patients fail to respond to these treatments. In contrast, understanding of the pathophysiology of akathisia remains limited. Considering the clinical traits of akathisia, it seems that the interaction of several neurotransmitter systems (for example, dopamine, acetylcholine, norepinephrine, serotonin, gama-aminobutyric acid (GABA, and neuropeptides) underlies its complex pathophysiology (10, 20). On the other hand, vitamin B6 is reported to have beneficial effects on movement disorders. It has been reported that it may have some benefits in reducing the severity of tardive dyskinesia symptoms among individuals with schizophrenia (21). Lerner and collegues in 2007 conducted A 26-week, double-blind, placebo-controlled trial using vitamin B6 (daily dose of 1200 mg) on 50 inpatients with DSM-IV diagnoses of schizophrenia or schizoaffective disorder and tardive dyskinesia (TD). They found that vitamin B6 appears to be effective in reducing symptoms of TD. However, the specific mechanisms by which vitamin B6 attenuates symptoms of TD are not clear (22).

The mechanism of action of vitamin B6 in clinical management of movement disorders is not clear. One possible explanation for this effect of vitamin B6 on akathisia may be related to its influence on neurotransmitters. Another mechanism might be related to its antioxidant and free radical scavenger property of pyridoxine (13, 23). Free radicals have been implicated in neuroleptic-induced movement disorders in Cadet *et al*. study who reported the beneficial effect of vitamin E in Tardive Dyskinesia (24). 

Considering the lower adverse effect of pyridoxine than propranolol and no significant difference between those results on antipsychotic-induced akathisia in present study, vitamin B6 may serve as a reasonable alternative option for patients with AIA. The results indicated that there wasn’t any significant difference between high or low dose of vitamin B6. However, more studies using higher and lower doses are needed for justifying the possible dose-response effect of this agent for ameliorating of antipsychotic-induced akathisia.

There was a limitation related to this study. The sample size (20 subjects per group) of present investigation was small. Therefore, the question of whether a larger sample size may have detected differences between the vitamin b6 versus propranolol group deserves further evaluations. 

## Conclusion

In conclusion, we found that vitamin B6 may serve as an alternative for propranolol in the management of antipsychotic induced akathisia in patients with schizophrenia. 
